# Supplementary material for: The Effects of Frequent Coffee Drinking on Female-Dominated Healthcare Workers Experiencing Musculoskeletal Pain and a Lack of Sleep
Source: J Pers Med. 2022 Dec 22;13(1):25. doi: 10.3390/jpm13010025 (PMC9866007; doi:10.3390/jpm13010025)
Supplement: Supplementary file 1 [file jpm-13-00025-s001.zip › jpm-2046654-supplementary.pdf]

Supplementary information

Table S1. the description of basic demography of 1615 participants

Survey variables	individuals	proportion (%)
**Gender**		
Female	1,314	81.36
Male	301	18.64
**Age**		
Less than or equal to 29	412	25.51
Between 29 and 38	433	26.81
Between 38 and 45	302	18.70
More than or equal to 45	468	28.98
**Marriage**		
Married	779	48.24
Other	836	51.76
**Having children**		
Parents	703	43.53
Not parents	912	56.47
**Education**		
Master's degree or above	297	18.39
University degree or below	1318	81.61
**Sleep duration per day**		
Less than 5 hours	63	3.90
Between 5 and 6 hours	563	34.86
Between 6 and 7 hours	719	44.52
Between 7 and 8 hours	232	14.37
More than 8 hours	38	2.35
**Coffee intake per day**		
More than 2 cups per day	26	1.61
2 cups per day	70	4.33
1 cup per day	556	34.43
Occasionally	678	41.98
Never	285	17.65
**Alcohol use**		
Alcohol use in a month	609	37.71
No alcohol use in a month	1006	62.29
**Exercise at least once a week**		
Yes	933	57.77
No	682	42.23

**Overtime work in a month**		
More than 80 hours	5	0.31
45–80 hours per month	54	3.34
Fewer than 45 hours	502	31.08
Seldom	1054	65.26
**Shift schedules**		
Irregular shifts	192	11.89
Regular shifts	196	12.14
Night shifts	166	10.28
Day shifts	1061	65.70
**Profession**		
Physicians	138	8.55
Nurses	613	37.96
Professional and technical personnel	283	17.52
Administrative staff	581	35.98
**Suffering from chronic diseases**		
Yes	638	39.51
No	977	60.49

Table S2. The description of sleep duration per day for all individuals

		Sleep duration per day					
Survey variables	N	< 5h	5~6h	6~7h	7~8h	> 8h	p
All individuals	1615	63	563	719	232	38	-
**Gender**							
Female	1,314	49	459	582	191	33	0.815[†]
Male	301	14	104	137	41	5	
**Age**							
Less than or equal to 29	412	11	157	170	60	14	0.114[†]
Between 29 and 38	433	23	154	175	70	11	
Between 38 and 45	302	12	104	144	36	6	
More than or equal to 45	468	17	148	230	66	7	
**Marriage**							
Married	779	26	247	373	119	14	0.016[†]
Other	836	37	316	346	113	24	
**Having children**							
Parents	703	26	224	336	105	12	0.063[†]
Not parents	912	37	339	383	127	26	

**Engaging in leisure activities with family/friends**							
Always	102	3	20	47	28	4	<.0001†
Often	498	11	161	238	74	14	
Sometime	765	31	272	351	98	13	
Seldom	238	17	106	79	30	6	
Never	12	1	4	4	2	1	
**Education**							
Master's degree or above	297	14	100	138	40	5	0.735†
University degree or below	1318	49	463	581	192	33	
**Coffee intake per day**							
More than 2 cups per day	26	2	17	4	2	1	<.0001†
2 cups per day	70	2	38	21	9	0	
1 cup per day	556	22	203	257	64	10	
Occasionally	678	23	231	318	94	12	
Never	285	14	74	119	63	15	
**Alcohol use**							
Alcohol use in a month	609	31	228	263	76	11	0.051†
No alcohol use in a month	1006	32	335	456	156	27	
**Exercise at least once a week**							
Yes	933	25	315	438	137	18	0.008†
No	682	38	248	281	95	20	
**Overtime work in a month**							
More than 80 hours	5	1	2	2	0	0	<.0001†
45–80 hours per month	54	4	26	22	1	1	
Fewer than 45 hours	502	29	199	183	83	8	
Seldom	1054	29	336	512	148	29	
**Shift schedules**							
Irregular shifts	192	11	87	67	24	3	<.0001†
Regular shifts	196	8	71	90	18	9	
Night shifts	166	6	54	62	33	11	
Day shifts	1061	38	351	500	157	15	
**Profession**							

Physicians	138	6	61	51	18	2	0.005†
Nurses	613	27	230	250	82	24	
Professional and technical personnel	283	11	81	147	40	4	
Administrative staff	581	19	191	271	92	8	
**Suffering from chronic diseases**							
Yes	638	30	238	275	84	11	0.142†
No	977	33	325	444	148	27	

N, individuals; †, Fisher exact test.

Table S3. The description of coffee intake per day for all individuals

		Coffee intake					
Survey variables	N	Never	Occ.	1 cup	2 cups	>2 cups	p
All individuals	1615	285	678	556	70	26	-
**Gender**							
Female	1,314	234	555	460	48	17	0.024†
Male	301	51	123	96	22	9	
**Age**							
Less than or equal to 29	412	131	198	73	9	1	<.0001†
Between 29 and 38	433	87	188	130	21	7	
Between 38 and 45	302	27	121	129	18	7	
More than or equal to 45	468	40	171	224	22	11	
**Marriage**							
Married	779	100	304	317	43	15	<.0001†
Other	836	185	374	239	27	11	
**Having children**							
Parents	703	81	265	306	37	14	<.0001†
Not parents	912	204	413	250	33	12	
**Engaging in leisure activities with family/friends**							
Always	102	20	34	42	4	2	0.052†
Often	498	84	186	190	29	9	
Sometime	765	126	358	244	27	10	
Seldom	238	51	98	75	9	5	
Never	12	4	2	5	1	0	

**Education**							
Master's degree or above	297	39	96	134	21	7	<.0001†
University degree or below	1318	246	582	422	49	19	
**Alcohol use**							
Alcohol use in a month	609	74	254	229	39	13	<.0001†
No alcohol use in a month	1006	211	424	327	31	13	
**Exercise at least once a week**							
Yes	933	146	378	344	51	14	0.002†
No	682	139	300	212	19	12	
**Overtime work in a month**							
More than 80 hours	5	0	2	1	1	1	0.142†
45–80 hours per month	54	12	20	15	4	3	
Fewer than 45 hours	502	84	219	167	21	11	
Seldom	1054	189	437	373	44	11	
**Shift schedules**							
Irregular shifts	192	31	85	66	7	3	0.137†
Regular shifts	196	38	99	48	9	2	
Night shifts	166	36	69	54	4	3	
Day shifts	1061	180	425	388	50	18	
**Profession**							
Physicians	138	24	52	45	13	4	0.001†
Nurses	613	124	275	189	16	9	
Professional and technical personnel	283	42	108	113	19	1	
Administrative staff	581	95	243	209	22	12	
**Suffering from chronic diseases**							
Yes	638	96	261	238	30	13	0.077†
No	977	189	417	318	40	13	

N, individuals; †, Fisher exact test.
